# Construction of the plasmid for expression of EGFP-M-IL-2(88Arg, 125Ala) fusion protein and the anti-tumor effects exerted by the fusion protein in HeLa-60 cells

**DOI:** 10.3892/ol.2021.12623

**Published:** 2021-03-10

**Authors:** Guangcan Shao, Dongmeng Qian, Haitao Wang, Zhiyong Yan, Ming Hu, Tongmei Wang, Bin Wang

Oncol Lett 9: 2729-2735, 2015; DOI: 10.3892/ol.2015.3125

Following the publication of the above article, an interested reader drew to the authors’ attention that the 24 h, A and the 48 h, C panels shown in Fig. 4 appeared to contain overlapping data. After having re-examined their original data, the authors have realized that this Figure was assembled incorrectly.

The corrected version of Fig. 4 is presented opposite. Note that this inadvertent error did not have a major impact on the results or the conclusions reported in this paper. Four of the named authors (Guangcan Shao, Dongmeng Qian, Ming Hu and Tongmei Wang) agree with this corrigendum; the other three authors proved to be uncontactable. These four authors would like to thank the Editor of *Oncology Letters* for allowing them the opportunity to publish this corrigendum; furthermore, they apologize to the readership of the Journal for any inconvenience caused.

## Figures and Tables

**Figure 4.**
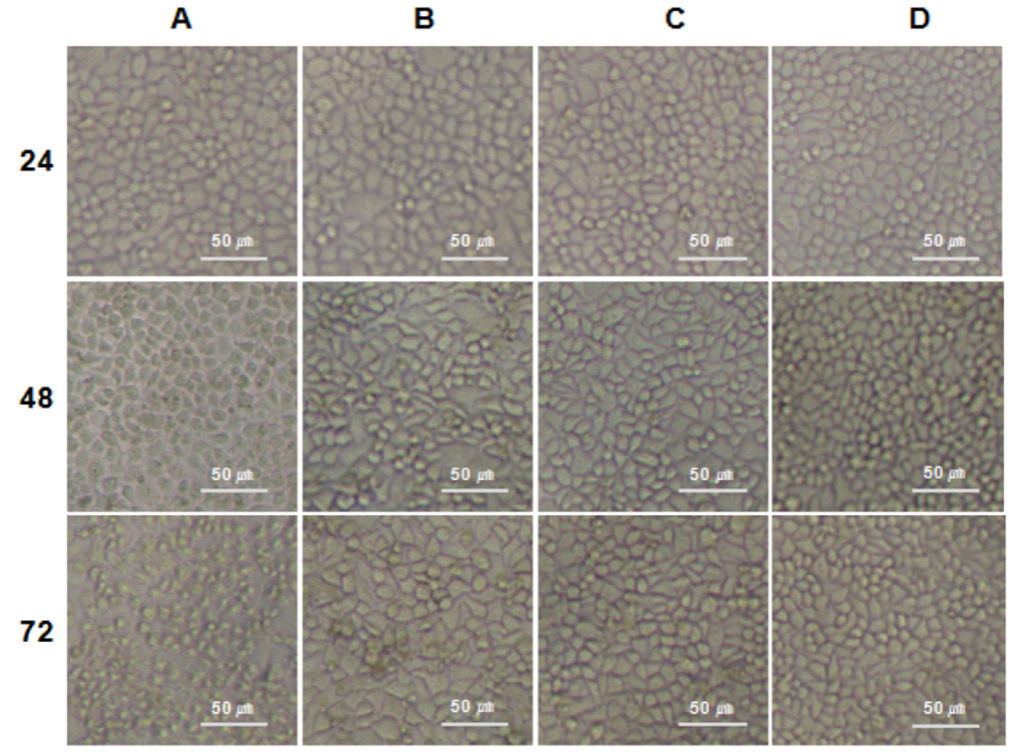
The morphology of various HeLa cell treatment groups were observed at 24, 48 and 72 h subsequent to transfection. (A) Cells transfected with pLEGFP-C1-M-IL-2(88Arg, 125Ala). (B) pLEGFP-C1-transfected cells. (C) Lipofectamine 2000-transfected cells. (D) non-transfected cells.

